# Randomized control trial to evaluate the effects of acute testosterone administration in men on muscle mass, strength, and physical function following ACL reconstructive surgery: rationale, design, methods

**DOI:** 10.1186/1471-2482-14-102

**Published:** 2014-12-06

**Authors:** Brian W Wu, Max Berger, Jonathan C Sum, George F Hatch, E Todd Schroeder

**Affiliations:** Keck School of Medicine, University of Southern California, Los Angeles, CA USA; >Biokinesiology and Physical Therapy, University of Southern California, Los Angeles, CA USA

**Keywords:** Anterior cruciate ligament, Rehabilitation, Testosterone, Orthopedic surgery

## Abstract

**Background:**

The anterior cruciate ligament (ACL) is one of four major ligaments in the knee that provide stability during physical activity. A tear in the ACL is characterized by joint instability that leads to decreased activity, knee dysfunction, reduced quality of life and a loss of muscle mass and strength. While rehabilitation is the standard-of-care for return to daily function, additional surgical reconstruction can provide individuals with an opportunity to return to sports and strenuous physical activity. Over 200,000 ACL reconstructions are performed in the United States each year, and rehabilitation following surgery is slow and expensive. One possible method to improve the recovery process is the use of intramuscular testosterone, which has been shown to increase muscle mass and strength independent of exercise. With short-term use of supraphysiologic doses of testosterone, we hope to reduce loss of muscle mass and strength and minimize loss of physical function following ACL reconstruction compared to standard-of-care alone.

**Methods/design:**

This study is a double-blinded randomized control trial. Men 18–50 years of age, scheduled for ACL reconstruction are randomized into two groups. Participants randomized to the testosterone group receive intramuscular testosterone administration once per week for 8 weeks starting 2 weeks prior to surgery. Participants randomized to the control group receive a saline placebo intramuscularly instead of testosterone. Lean mass, muscle strength and physical function are measured at 5 time points: 2 weeks pre-surgery, 1 day pre-surgery, and 6, 12, 24 weeks post-surgery. Both groups follow standard-of-care rehabilitation protocol.

**Discussion:**

We believe that testosterone therapy will help reduce the loss of muscle mass and strength experienced after ACL injury and reconstruction. Hopefully this will provide a way to shorten the rehabilitation necessary following ACL reconstruction. If successful, testosterone therapy may also be used for other injuries involving trauma and muscle atrophy.

**Trial registration:**

NTC01595581, Registration: May 8, 2012

## Background

The anterior cruciate ligament is one of four major ligaments in the knee and provides stability during physical activity. A tear in the ligament is characterized by joint instability that leads to decreased activity, knee dysfunction, and reduced quality of life [1]. The initial knee injury is associated with rapid loss of muscle mass and strength, which can be regained with structured rehabilitation [1]. While rehabilitation is the standard-of-care for return to daily function, additional surgical reconstruction can provide individuals with an opportunity to return to sports and strenuous physical activity [2]. However, surgery will initially add more trauma to the knee and prolong rehabilitation. Together, these components can lead to a slow and expensive recovery [3]. Because there are at least 200,000 anterior cruciate ligament (ACL) reconstructions performed each year in the United States [1], it will be beneficial to improve the recovery process. One possible method may be the use of intramuscular testosterone, which has been shown to increase muscle mass and strength independent of exercise [4, 5]. Inducing an anabolic environment with a short course of testosterone may be an efficient method to improve recovery.

Muscle mass is dependent on the ratio of muscle protein synthesis (anabolism) to muscle protein breakdown (catabolism). Trauma from surgery and limited movement of the knee result in atrophy and breakdown due to a catabolic state and reduced anabolism of skeletal muscle proteins. Furthermore, ACL injuries can result in weeks of immobilization of the knee, and animal studies have shown that anabolism decreases after as few as 6 hours of cast immobilization [6]. The combination of surgery, the initial knee injury, and the associated limited movement contribute to the delayed and intensive rehabilitative process.

Testosterone increases myofibrillar protein synthesis and promotes anabolism of muscle tissue. It also modulates the activity of immune, fibroblast, and myogenic precursor cells, which are all involved in muscle regeneration. Furthermore, testosterone administration increases lean tissue and maximal voluntary strength in a dose-dependent manner [5]. Animal models have shown that exogenous testosterone aids in muscle regeneration following several types of injury, such as crush injuries, venom-induced muscle injury, muscle disuse atrophy, and injury following muscle graft surgery [7]. With testosterone, successful regeneration of healthy mouse muscle can occur within 2–3 weeks of injury where muscle strength can return to pre-injured values [7].

Testosterone may also induce muscle growth via growth hormone (GH) and insulin-like growth factor 1 (IGF-1) [7, 8]. IGF-1 can stimulate muscle protein synthesis and satellite cell activity and anabolic steroids increase both circulating IGF-1 and muscle mRNA expression of *IGF*-*1*. Akt (protein kinase B) is a signaling mediator of IGF-1 that can regulate muscle mass. However, it is also known that testosterone can work outside of the Akt signaling pathway in order to maintain skeletal muscle hypertrophy [7, 9]. We are studying key regulatory proteins such as Akt1, mTOR, and FOXO3a, which have not been studied in concurrent states of muscle trauma and testosterone administration.

**Figure 1:**
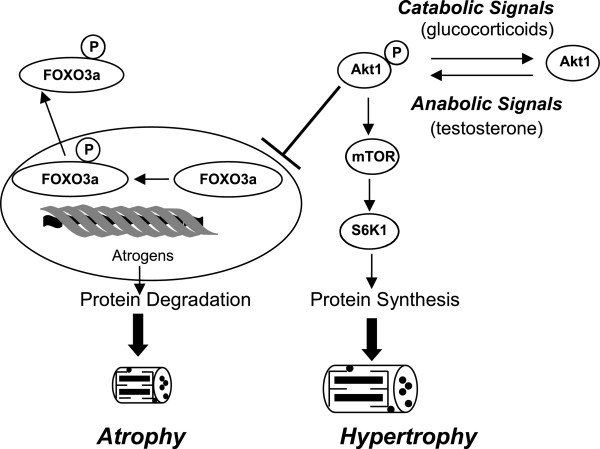
**Akt1 signaling and control of skeletal muscle hypertrophy and atrophy.** Anabolic signals (e.g. testosterone) initiate phosphorylation (P) of Akt1, which activates protein synthesis via the mTOR pathway. At the same time, Akt1(P) inactivates FOXO3a by phosphorylation and facilitates translocation of FOXO3a out of the nucleus, resulting in inhibition of the atrophy-related genes (atrogens), and thereby decreasing protein degradation. On the other hand, catabolic stimuli (e.g. glucocorticoids) dephosphorylate and thereby inactivate the Akt1 protein. Inactivation of the Akt1 protein allows expression of *FOXO3a* in the nucleus and subsequent activation of the atrogens, resulting in protein degradation. (Figure adapted from G.A. Nader) [10].

There is evidence that the Akt pathway regulates gene transcription through the inactivation of a group of transcription factors (FOXOs) located in the nucleus [10, 11]. FOXO3a increases the transcription of atrophy related genes (atrogens), decreases protein degradation, and results in muscle atrophy. Phosphorylation of FOXO3a by phosphorylated Akt1 inactivates the transcription factor, releasing it from DNA and resulting in translocation of the inactive FOXO3a to the cytosol [11]. Phosphorylated Akt1 will also activate the mTOR pathway to stimulate protein synthesis and result in hypertrophy [11]. Therefore, the Akt pathway may regulate both skeletal muscle protein synthesis and degradation by different but complimentary mechanisms in response to testosterone (Figure 1). By analyzing muscle tissue following an ACL rupture with or without testosterone administration, we can learn more about the mechanism through which muscle adapts to trauma and how testosterone affects this process. Therefore, we may learn more about testosterone’s mechanism of action in skeletal muscle during the catabolic stimulus of bedrest and surgery.

In addition to understanding the possible mechanisms of testosterone, there may be improved recovery time as measured by questionnaires and function tests. In a study using the Tegner Activity Scale [12], a questionnaire developed to describe a patient’s physical activity, 60% of participants who received ACL reconstruction did not return to pre-injury activity level within 2 years [1]. In another study where supraphysiologic doses of testosterone were given to older men undergoing knee replacement, the physical function as measured by the Functional Independence Measure ability to stand was significantly higher by 30% in patients who received testosterone. Similarly, we hope to improve recovery following ACL reconstruction using supraphysiologic doses of testosterone. Specifically, we hope to reduce the loss of muscle mass and strength and minimize loss of physical function. Should acute testosterone improve rehabilitation following ACL surgery, it may be possible to apply the same concept to other injuries that involve muscle atrophy.

### Objectives

The overall objective of this study is to determine if 8 weeks of testosterone first administered 2 weeks prior to surgery, can improve the outcome of ACL reconstruction. Specifically, we will evaluate the effect of testosterone administration on lean muscle mass and strength, physical function, clinical outcomes, and skeletal muscle myogenic regulators following ACL reconstructive surgery. We hypothesize that testosterone will minimize the reductions or potentially increase muscle mass and strength following surgery and may hasten a patient’s return to physical activity. If testosterone improves recovery after ACL surgery, the same treatment may be used for other injuries that involve trauma and muscle atrophy. Furthermore, this study will examine the effect of trauma with or without testosterone on myogenic regulators in muscle tissue taken during ACL surgery—providing possible mechanistic insights for the clinical outcomes.

## Methods

### Study design

Men, 18–50 years of age, scheduled for ACL reconstructive surgery will be randomized to one of two groups. Although most ACL tears occur in women [2], this study will be limited to men due to the gender-related side effects of testosterone. According to our power analysis, we aim to have at least 7 patients in each group after recruiting and screening approximately 70 patients. The dosing is based on a previous study using supraphysiological dose of testosterone for knee replacement surgery [13]. Intramuscular testosterone cypionate will be used because it has been more widely studied and is less expensive than the gel or patch forms of testosterone. Group 1 will receive testosterone administration, one weekly 200 mg dose intramuscularly of testosterone in sesame oil, for 8 continuous weeks beginning 2 weeks prior to surgery and standard-of-care rehabilitation following surgery. Group 2 will follow the same dosing and rehabilitation schedule but receive a saline placebo instead of testosterone. The dose will be given intramuscularly to minimize systemic side effects; however, we expect the total systemic levels of testosterone to increase to an average of 1200 ng/dl. Lean mass, muscle strength, and physical function will be measured 2 weeks prior to surgery, 1 day prior to surgery, and 6, 12, and 24 weeks following surgery. By administering the dose 2 weeks prior to surgery, there will be sufficient time to develop an anabolic state before the catabolic stimulus of surgery. The dose will continue for 8 weeks to try to maintain muscle mass when there is limited movement and endogenous anabolic stimulus. After 6 weeks from the surgery, testosterone will no longer be administered because KOOS function and sports score appear to stabilize after this time [1]. The study will be a double-blinded experiment.

Patients will follow a standard rehabilitation protocol [14] customized by an orthopedic physician. Rehabilitation will begin shortly after surgery and will be supervised by a licensed physical therapist. The protocol includes goals for range of motion, muscle function, and functional performance at different post-operative stages. The goals for each stage must be met before patient progresses to the next stage and the amount of exercises will be closely monitored by physical therapists and the graduate student. A quicker progression is expected in patients assigned to standard-of-care rehabilitation with testosterone. If any pain or swelling slows progression, the patient will see the treating clinician.

The study will be conducted at the University of Southern California (USC), Keck School of Medicine in Los Angeles, California and was approved by the Institutional Review Board of the University of California (HS-11-00649).

### Selection and randomization of subjects

Male participants, 18–50 years of age, who present to the orthopedic surgeon with recent knee trauma will be screened for eligibility. Eligible patients will have had rotational trauma to a previously uninjured knee within the preceding 8 months, ACL insufficiency as determined by clinical examination (positive pivot shift and/or positive Lachmann test), and a score of 5 to 9 on the Tegner Activity Scale (TAS) [12] before the injury (scores range from 1 to 10, with a score of 5 indicating participation in recreation sports, and a score of 9 indicating participation in competitive sports on a nonprofessional level).

All eligible participants (see Table 1) will receive information about the trial orally and in writing. After signing a written informed consent, they will be randomly assigned to undergo either standard-of-care structured rehabilitation with testosterone administration or standard-of-care structured rehabilitation with placebo. An investigator not involved in the randomization procedure will prepare sequentially numbered, opaque, sealed envelopes containing the assigned interventions to ensure randomness which will then be provided to the research pharmacist. The study is a double blind study. Only the research pharmacist will know which patient is receiving study drug or placebo. The surgeon, PI, study team, and physical therapist will not know which study arm the patient was randomized. The protocol for rehabilitation will remain the same for all patients.

**Table 1:** **Inclusion and exclusion criteria**

Inclusion criteria	Exclusion criteria
Eligible participants will have had:	• Previous major knee injury or knee surgery
• A complete ACL tear as visualized on MRI	• Associated posterior cruciate ligament (PCL) or medical collateral ligament (MCL) injury grade III
° The ACL injury can be either “isolated” or combined with one or several of the following injuries visualized on MRI and/or arthroscopy:	• Concomitant severe injury to contra-lateral knee
▪ A meniscus tear that is either left untreated or treated with a partial resection	• Injury to the lateral/posterolateral ligament complex with significantly increased laxity
▪ A small, stable meniscus tear treated with fixation, but with the fixation not interfering with the rehabilitation protocol	• Unstable longitudinal meniscus tear that requires repair and where the following postoperative treatment (e.g. bracing and limited range of motion) interferes with the rehabilitation protocol
▪ Cartilage changes verified on MRI with an arthroscopically determined intact surface.	• Bi-compartmental extensive meniscus resections
• A radiographic examination with normal joint status or combined with either one of the following findings:	• Cartilage injury representing a full thickness loss down to bone
° A small-avulsed fragment located laterally, usually described as a Segond fracture, JSN grade 1 or osteophytes grade 1 as determined by the OARSI atlas[15]	• Total rupture of MCL/LCL as visualized on MRI.
	• History of deep vein thrombosis (DVT) or a disorder of the coagulative system
	• Claustrophobia
	• Prior or current use of anabolic steroids
	• General systemic disease affecting physical function
	• Chromosomal disorders
	• Medications that interfere with testosterone production or function, including but not limited to 5α-reductase inhibitors
	• Any other condition or treatment interfering with the completion of the trial

### Study agent administration and safety

Although the study involves a very short course of testosterone, it may still have side effects on the endocrine systems; and therefore, common markers of endocrine function are monitored. Blood analysis will be performed at USC. Pituitary hormones (LH and FSH), prostate-specific antigen (PSA), liver enzymes, hematocrit, and blood pressure are measured at two weeks preoperatively, 1 day preoperatively, and 2, 3, 6, 12, and 24 weeks postoperatively.

### Orthopedic surgery

Surgery will be performed for all eligible patients within 8 months after the injury by a licensed orthopedic surgeon. Surgery will be performed while the patient is under general anesthesia. Meniscal surgery will be carried out as needed, followed by ACL reconstruction. All surgeons have similar success rates for ACL reconstruction.

### Rehabilitation

Rehabilitation will follow a standard guideline [16] under the supervision of a licensed physical therapist and the participant’s doctor.

### Muscle biopsies

During surgery, vastus lateralis muscle tissue will be obtained. The tissue will then be examined by q-PCR for changes in the gene expression of *Akt*-*1*, *mTOR*, and *FOXO3a* [17–21]. In tissues that have received 2 weeks of testosterone, we expect to see up-regulated expression of *Akt*-*1* and *mTOR*, and down-regulated expression of *FOXO3a*. Akt-1 phosphorylation will also be detected by western blotting, and should be higher in patients receiving testosterone.

### Clinical and laboratory evaluations

The patient will be tested on multiple occasions. All testing takes place at the USC Clinical Exercise Research Center (CERC). Information will be collected 2 weeks prior to surgery, followed by measurements 1 day prior to surgery, and 2, 6, 12, and 24 weeks after surgery. Surgery will be performed on an arranged date with the orthopedic surgeon. Physical therapy will follow a pre-defined protocol with a physical therapist.

Table 2 outlines the timeline used for the study including the dates of each visit and what takes place on each visit.

**Table 2:** **Study calendar**

Timeline	Drug delivery	Blood collection	BC testing	PF testing	Questionnaires
2 weeks prior to surgery	X	X	X	X	X
1 week prior to surgery	X				
1 day prior to surgery	X	X	X	X	X
1 week post-surgery	X				
2 weeks post-surgery	X	X			
3 weeks post-surgery	X				
4 weeks post-surgery	X				
5 weeks post-surgery	X				
6 weeks post-surgery		X	X	X	X
12 weeks post-surgery		X	X	X	X
24 weeks post-surgery		X	X	X	X

BC = Body Composition, PF = Physical function.

#### Drug delivery

Either testosterone or placebo will be injected intramuscularly once per week for 8 weeks, starting 2 weeks prior to surgery and ending 5 weeks post-surgery.

#### Blood draw

Blood will be collected to monitor any adverse effects on the participant’s health.

#### Body composition testing by bioelectrical impedance

The Biospace InBody 520 [22] device measures body composition as the participant stands on a scale-like device while grasping two handles; one in each hand. The device works by sending a very low-voltage electrical signal through the body to determine water content, body fat percentage, and lean (muscle) mass.

#### Body composition testing by DXA

We will also measure the participant’s lean mass and body fat percentage by whole-body dual-energy x-ray absorptiometry [22–24] (DXA), which is more sensitive to small changes in body composition than bioelectrical impedance. The subject will have DXA scans throughout the study. The DXA works by passing low energy x-rays through the subject’s body. The x-ray is absorbed differently by muscle tissue than fat tissue which allows the device to differentiate the amount of lean mass and fat mass in the participant’s body.

#### Physical function tests

At 2 weeks prior to surgery, 1 day prior to surgery, and 6, 12, and 24 weeks after surgery, tests of maximal muscle strength using a Cybex dynamometer [25–27] will be performed on the subject’s unaffected and affected leg. In addition, knee stability and flexibility tests of the affected leg are typically performed during rehabilitation. During later stages of rehabilitation, tests may include the single leg squat, single leg hop, X-hop, triple hop, and timed hop tests [19, 28–31].

#### Questionnaires

The subject will be asked to complete the Knee Injury and Osteoarthritis Outcome Score [32, 33] (KOOS), a commonly used test to measure progression after ACL reconstruction, and the Tegner Activity Scale [12, 34–37] to determine physical activity level.

### Statistical data analysis

Descriptive statistics will be performed for participant characteristics as well as baseline testosterone, strength, and body composition. Comparisons across groups will be made for each measurement (t-test) to determine if bias exists between the randomized groups. If bias does exist, these variables will be included as covariates in the primary analysis. The raw data (pre- and post-test) will be plotted to determine if outliers exist. Because the sample size is small if outliers are found, transformations of the data (e.g. log transformation) will be attempted. If this is not feasible, results will be compared using non-parametric analogs to the parametric tests listed below. Intent to treat analyses for specific hypotheses are listed below. In addition, effect sizes will be computed with Cohen’s D to determine the clinical relevance of the results and assist in powering further research. Analyses will be performed using SPSS for all analyses with α = 0.05. If there is any missing data, the analyses can be performed with the SPSS multiple imputation procedure.

Intent to treat will be tested by comparing changes in lean mass and strength using ANCOVA models, with group adjusting for baseline values. KOOS and TAS scores will be analyzed by ANCOVA models. Physical function tests scores on the Lachman and pivot shift tests will be analyzed by χ^2^ test. The changes in gene expression of *Akt1*, *mTOR*, and *FOXO3a* will be compared by ANCOVA models.

#### Power analysis

Sample size estimates were computed with Nquery (v4) for α = 0.05 and 1-β = 0.80. Preliminary data was taken from a study that provided 12 weeks of oxandrolone/day in 32 healthy 60–87 year old men [24]. Effects sizes between changes in means from baseline to 12 weeks for lean body mass and maximal voluntary strength were used to estimate the changes in mass and strength for Aim 1. The effect size was 2.0 for mass and 2.03 for strength. For similar effects, a minimum of 6 men would be needed for each group. Assuming a 20% attrition rate [1] and similarly very large effect, our sample of 7 per group should be sufficient to see statistically significant effects.

## Discussion

In this study, we are testing the hypothesis that standard-of-care rehabilitation with the addition of supraphysiologic doses of testosterone for 8 weeks will augment muscle mass, strength, and physical function following ACL reconstructive surgery compared to standard rehabilitation alone.

### Use of testosterone

Testosterone is the principal male sex hormone and an anabolic steroid. It is essential for healthy males. In men, testosterone plays a key role in the development of male reproductive tissues and promotes secondary sexual characteristics such as increased muscle, bone mass, and the growth of body hair. In young healthy male subjects similar to those used in our study, supraphysiologic doses of testosterone have been shown to increase fat-free mass, muscle size and strength independent of exercise [4]. This effect was even greater when testosterone was used in conjunction with strength training. Additionally, testosterone likely causes some of its effects, such as muscle strength, in a dose dependent manner [38].

While few studies have used supraphysiologic doses of testosterone, the ideal dosing for adjunctive therapy with ACL surgery is not known. Because our patients will not necessarily be hypogonadal, as is typical for testosterone replacement studies, there is a risk that our dose may not be large enough to have a significant influence on muscle mass and strength. Alternatively, a dose that is too high (>600 mg/wk) [38] or given for long duration (6 months) [39] may have deleterious effects by increasing risk of cardiovascular disease. However, we believe that our chosen dose regimen will be safe and successful as it is based on the current literature and the expertise of our study team [5, 13].

The potential detrimental cardiovascular side effects of testosterone have recently been featured in both the literature and the media [40]. However, these studies focus primarily on testosterone replacement therapy for an extended duration and are mainly in hypogonadal men and therefore are not as applicable to our current study. We do not anticipate any negative side effects of the testosterone treatment, but will be monitoring all patients closely for any potential complications.

### Study participants

In our study, we will use young healthy males aged 18–50 with recent acute ACL tears. This demographic will be used in an attempt to isolate the effect of testosterone and minimize any confounding factors such as age, comorbidities and sex. Women will not be used in this initial study due to the gender related side effects of testosterone. Additionally, the results showing the ability of supraphysiologic doses of testosterone to augment muscle size and strength were reported for young healthy males [38]. However, we do not believe that the benefits of testosterone on rehabilitation are isolated to this demographic alone. If this study shows significant results, the next step would be to expand our age range and conduct studies with other atrophy-related surgeries such as total knee arthoplasty.

### Limitations

Testosterone therapy is currently a controversial topic but previous studies have shown positive results [8, 41–48]. It may have been ideal to personalize the supraphysiologic doses according to baseline for each participant. However, for ease of administration and future research viability, we attempted to standardize this value. It may be interesting to note variability of the peak testosterone values and results for those participants. Similarly, there is a large age range of participants and studies have shown differences in testosterone level as early as 40 years of age [44, 48–52]. However, additional studies show that the effects in males will still exist with supraphysiologic doses [48, 53, 54] and we expect an effect to be measured independent of age or race.

Recruitment of participants who meet all inclusion criteria will be challenging. Although we are working with multiple orthopedic surgeons, there are a number of challenges in recruiting patients with ACL injury into randomized clinical trials [55]. It will likely be necessary to recruit and screen approximately 70 participants -- 5 times as many potential participants as our power analysis indicates to demonstrate significant differences.

Additionally, while we aim to work with as few physical therapists as possible and are following a standardized protocol, there are differences in the techniques and progression of individual therapists. We intend to monitor progress of individual participants as standardized as possible and to also focus on the 24 week result as the primary outcome time point.

### Relevance for investigating the effects of testosterone on rehabilitation

The present study will contribute to the field of rehabilitation following invasive surgery with respect to understanding an innovative approach towards treating injury induced muscle atrophy. By examining the effects of testosterone following surgery as an adjunct therapy, we can observe the effects on multiple translational levels including clinical outcomes, quality of life, and mechanisms. The results of the study will help provide the framework for additional large scale and multi-centered investigations to optimize rehabilitation in surgeries that affect many persons and injuries.

Because many patients do not return to pre-surgical levels of activity and strength [1, 29, 56], continued research is necessary to study muscle atrophy, a critical component of this poor return. Furthermore, these effects are often compounded in elite athletes [28, 57] and although this present study does not focus on these patients, they are a crucial group that may benefit from such research. Ideally, such methods for targeting muscle atrophy should be applicable across gender, age, and injury type. Future and vigorous research will be required to test such hypotheses, as testosterone levels play a critical role in overall health.

Testosterone has never been studied in the age range of our patients for therapy following injuries associated with muscle atrophy. We believe that testosterone will improve the outcome of ACL reconstruction and augment muscle mass, strength, and physical function compared to standard-of-care alone. If our study demonstrates that acute testosterone improves rehabilitation following ACL reconstruction, it may be possible to apply the same concept to other injuries that involve muscle atrophy. With almost every orthopedic injury and accompanying surgery, there is a disuse muscle atrophy that occurs that will ultimately lengthen the period of rehabilitation needed to get back to full strength [6]. We believe that testosterone therapy will help prevent this loss of muscle mass and can shorten the length of rehabilitation needed in these patients. In addition, by analyzing muscle tissue following an ACL rupture with or without testosterone administration, we can learn more about the mechanism through which muscle adapts to trauma and how testosterone affects this process. Therefore, we may learn more about testosterone’s mechanism of action in skeletal muscle during the catabolic stimulus of bedrest and surgery.

A unique aspect of our proposed adjunct therapy to ACL reconstruction is its clinical feasibility. Testosterone therapy is easily added to the standard-of-care rehabilitation with little additional time or effort required by the patient. The testosterone injections can be administered at routine preoperative and postoperative visits, with few extra office visits necessary. This would allow many additional patients to have access to the therapy regardless of time constraints.

## Conclusion

This study will contribute to the understanding of skeletal muscle adaptations to trauma and surgery, and how testosterone can affect this process. We believe that testosterone will augment muscle mass and improve rehabilitation times following ACL reconstructive surgery and quality of life for patients with this injury. In the future, we hope that the principles of testosterone therapy following injury and disuse atrophy can be expanded to a larger cohort of patients and a more diverse range of injuries and surgeries. The results from this study will allow for larger scale studies investigating the optimization of testosterone therapy in rehabilitation for various types of injuries.

## Authors’ information

BW, JS, RH, TS provided conception, design, trial protocol and initiation of the project; BW is the study coordinator, providing supervision of physical function testing and specimen collection; MB and BW patient recruitment, data collection and entry, drafted and finalized the manuscript. All authors have read and approved the final manuscript.

## Abbreviations

ACL: Anterior cruciate ligament
GH: Growth hormone
IGF-1: Insulin-like growth factor 1
KOOS: Knee Injury and Osteoarthritis Outcome Score
TAS: Tegner Activity Score
PCL: Posterior cruciate ligament
MCL: Medial collateral ligament
LH: Luteinizing hormone
FSH: Follicle stimulating hormone
PSA: Prostate specific antigen
CERM: USC Clinical Exercise Research Center
BC: Body composition
PF: Physical function
DXA: Dual Energy X-ray Absorptiometry
SPSS: Statistical Package for the Social Sciences
ANCOVA: Analysis of covariance.

## References

[1] Frobell RB, Roos EM, Roos HP, Ranstam J, Lohmander LS (2010). A randomized trial of treatment for acute anterior cruciate ligament tears. N Engl J Med.

[2] Spindler KP, Wright RW (2008). Anterior Cruciate Ligament Tear. N Eng J Med.

[3] Arangio GA, Chen C, Kalady M, Reed JF (1997). Thigh muscle size and strength after anterior cruciate ligament reconstruction and rehabilitation. J Orthop Sports Phys Ther.

[4] Bhasin S, Storer TW, Berman N, Callegari C, Clevenger B, Phillips J, Bunnell TJ, Tricker R, Shirazi A, Casaburi R (1996). The effects of supraphysiologic doses of testosterone on muscle size and strength in normal men. N Engl J Med.

[5] Schroeder ET, Terk M, Sattler FR (2003). Androgen therapy improves muscle mass and strength but not muscle quality: results from two studies. Am J Physiol Endocrinol Metab.

[6] Marimuthu K, Murton AJ, Greenhaff PL (2011). Mechanisms regulating muscle mass during disuse atrophy and rehabilitation in humans. J Appl Physiol (Bethesda, Md : 1985).

[7] White JP, Baltgalvis K, Sato S, Wilson LB, Carson JA (2009). Effect of nandrolone decanoate administration on recovery from bupivacaine-induced muscle injury. J Appl Physiol (Bethesda, Md: 1985).

[8] Serra C, Bhasin S, Tangherlini F, Barton ER, Ganno M, Zhang A, Shansky J, Vandenburgh HH, Travison TG, Jasuja R, Morris C (2011). The role of GH and IGF-I in mediating anabolic effects of testosterone on androgen-responsive muscle. Endocrinology.

[9] Hourdé C, Jagerschmidt C, Clément-Lacroix P, Vignaud A, Ammann P, Butler-Browne GS, Ferry A (2009). Androgen replacement therapy improves function in male rat muscles independently of hypertrophy and activation of the Akt/mTOR pathway. Acta Physiologica (Oxford, England).

[10] Nader GA (2005). Molecular determinants of skeletal muscle mass: getting the "AKT" together. Int J Biochem Cell Biol.

[11] Sandri M, Sandri C, Gilbert A, Skurk C, Calabria E, Picard A, Walsh K, Schiaffino S, Lecker SH, Goldberg AL (2004). Foxo transcription factors induce the atrophy-related ubiquitin ligase atrogin-1 and cause skeletal muscle atrophy. Cell.

[12] Tegner Y, Lysholm J (1985). Rating systems in the evaluation of knee ligament injuries. Clin Orthop Relat Res.

[13] Amory JK, Chansky HA, Chansky KL, Camuso MR, Hoey CT, Anawalt BD, Matsumoto AM, Bremner WJ (2002). Preoperative supraphysiological testosterone in older men undergoing knee replacement surgery. J Am Geriatr Soc.

[14] Oiestad BE, Engebretsen L, Storheim K, Risberg MA (2009). Knee osteoarthritis after anterior cruciate ligament injury: a systematic review. Am J Sports Med.

[15] Hochberg MC, Altman RD, Brandt KD, Clark BM, Dieppe PA, Griffin MR, Moskowitz RW, Schnitzer TJ (1995). Guidelines for the medical management of osteoarthritis. Part I Osteoarthritis of the hip American College of Rheumatology. Arthritis Rheum.

[16] Wilk KE, Macrina LC, Cain EL, Dugas JR, Andrews JR (2012). Recent advances in the rehabilitation of anterior cruciate ligament injuries. J Orthop Sports Phys Ther.

[17] Jorge MLMP, de Oliveira VN, Resende NM, Paraiso LF, Calixto A, Diniz ALD, Resende ES, Ropelle ER, Carvalheira JB, Espindola FS, Jorge PT, Geloneze B (2011). The effects of aerobic, resistance, and combined exercise on metabolic control, inflammatory markers, adipocytokines, and muscle insulin signaling in patients with type 2 diabetes mellitus. Metab Clin Exp.

[18] Wolsk E, Mygind H, Grøndahl TS, Pedersen BK, van Hall G (2010). IL-6 selectively stimulates fat metabolism in human skeletal muscle. Am J Physiol Endocrinol Metab.

[19] Barker T, Leonard SW, Hansen J, Trawick RH, Ingram R, Burdett G, Lebold KM, Walker JA, Traber MG (2009). Vitamin E and C supplementation does not ameliorate muscle dysfunction after anterior cruciate ligament surgery. Free Radic Biol Med.

[20] Wilborn CD, Taylor LW, Greenwood M, Kreider RB, Willoughby DS (2009). Effects of different intensities of resistance exercise on regulators of myogenesis. J Strength Cond Res.

[21] Chen YW, Zhao P, Borup R, Hoffman EP (2000). Expression profiling in the muscular dystrophies: identification of novel aspects of molecular pathophysiology. J Cell Biol.

[22] Jensky-Squires NE, Dieli-Conwright CM, Rossuello A, Erceg DN, McCauley S, Schroeder ET (2008). Validity and reliability of body composition analysers in children and adults. Br J Nutr.

[23] Schroeder ET, He J, Yarasheski KE, Binder EF, Castaneda-Sceppa C, Bhasin S, Dieli-Conwright CM, Kawakubo M, Roubenoff R, Azen SP, Sattler FR (2011). Value of measuring muscle performance to assess changes in lean mass with testosterone and growth hormone supplementation. Eur J Appl Physiol.

[24] Schroeder ET, Zheng L, Yarasheski KE, Qian D, Stewart Y, Flores C, Martinez C, Terk M, Sattler FR (2004). Treatment with oxandrolone and the durability of effects in older men. J Appl Physiol.

[25] Brown K, Swank AM, Quesada PM, Nyland J, Malkani A, Topp R (2010). Prehabilitation versus usual care before total knee arthroplasty: A case report comparing outcomes within the same individual. Physiother Theory Pract.

[26] Meier WA, Marcus RL, Dibble LE, Foreman KB, Peters CL, Mizner RL, LaStayo PC (2009). The long-term contribution of muscle activation and muscle size to quadriceps weakness following total knee arthroplasty. J Geriatr Phys Ther.

[27] Heijne A, Werner S (2007). Early versus late start of open kinetic chain quadriceps exercises after ACL reconstruction with patellar tendon or hamstring grafts: a prospective randomized outcome study. Knee Surg, Sports Traumatol, Arthroscopy : Off J ESSKA.

[28] Myer GD (2011). Utilization of Modified NFL Combine Testing to Identify Functional Deficits in Athletes Following ACL Reconstruction. J Orthopaedic Sports Physical Therapy.

[29] Cimino F, Volk BS, Setter D (2010). Anterior cruciate ligament injury: diagnosis, management, and prevention. Am Fam Physician.

[30] Hohmann E, Tetsworth K, Hohmann S, Bryant AL (2010). Anabolic steroids after total knee arthroplasty. A double blinded prospective pilot study. J Orthop Surg Res.

[31] Samuelsson K, Andersson D, Karlsson J (2009). Treatment of anterior cruciate ligament injuries with special reference to graft type and surgical technique: an assessment of randomized controlled trials. Arthroscopy: J Arthrosc Relat Surg: Off Publ Arthrosc Assoc N Am Int Arthrosc Assoc.

[32] Hambly K, Griva K (2010). IKDC or KOOS: which one captures symptoms and disabilities most important to patients who have undergone initial anterior cruciate ligament reconstruction?. Am J Sports Med.

[33] Roos EM, Roos HP, Lohmander LS, Ekdahl C, Beynnon BD (1998). Knee Injury and Osteoarthritis Outcome Score (KOOS)–development of a self-administered outcome measure. J Orthop Sports Phys Ther.

[34] Lind M, Menhert F, Pedersen AB (2009). The first results from the Danish ACL reconstruction registry: epidemiologic and 2 year follow-up results from 5,818 knee ligament reconstructions. Knee Surg Sports Traumatol Arthrosc.

[35] Kessler MA, Behrend H, Henz S, Stutz G, Rukavina A, Kuster MS (2008). Function, osteoarthritis and activity after ACL-rupture: 11 years follow-up results of conservative versus reconstructive treatment. Knee Surg Sports Traumatol Arthrosc.

[36] Maletis GB, Cameron SL, Tengan JJ, Burchette RJ (2007). A prospective randomized study of anterior cruciate ligament reconstruction: a comparison of patellar tendon and quadruple-strand semitendinosus/gracilis tendons fixed with bioabsorbable interference screws. Am J Sports Med.

[37] Spindler KP, Kuhn JE, Freedman KB, Matthews CE, Dittus RS, Harrell FE (2004). Anterior cruciate ligament reconstruction autograft choice: bone-tendon-bone versus hamstring: does it really matter? A systematic review. Am J Sports Med.

[38] Bhasin S, Woodhouse L, Casaburi R, Singh AB, Bhasin D, Berman N, Chen X, Yarasheski KE, Magliano L, Dzekov C, Dzekov J, Bross R, Phillips J, Sinha-Hikim I, Shen R, Storer TW (2001). Testosterone dose–response relationships in healthy young men. Am J Physiol Endocrinol Metab.

[39] Basaria S, Coviello AD, Travison TG, Storer TW, Farwell WR, Jette AM, Eder R, Tennstedt S, Ulloor J, Zhang A, Choong K, Lakshman KM, Mazer NA, Miciek R, Krasnoff J, Elmi A, Knapp PE, Brooks B, Appleman E, Aggarwal S, Bhasin G, Hede-Brierley L, Bhatia A, Collins L, LeBrasseur N, Fiore LD, Bhasin S (2010). Adverse events associated with testosterone administration. N Engl J Med.

[40] Finkle WD, Greenland S, Ridgeway GK, Adams JL, Frasco MA, Cook MB, Fraumeni JF, Hoover RN (2014). Increased risk of non-fatal myocardial infarction following testosterone therapy prescription in men. PLoS One.

[41] Fu R, Liu J, Fan J, Li R, Li D, Yin J, Cui S (2010). Novel evidence that testosterone promotes cell proliferation and differentiation via G protein-coupled receptors in the rat L6 skeletal muscle myoblast cell line. J Cell Physiol.

[42] O'Connell MDL, Roberts SA, Srinivas-Shankar U, Tajar A, Connolly MJ, Adams JE, Oldham JA, Wu FCW (2011). Do the effects of testosterone on muscle strength, physical function, body composition, and quality of life persist six months after treatment in intermediate-frail and frail elderly men?. J Clin Endocrinol Metab.

[43] Saad F, Gooren LJ (2011). The role of testosterone in the etiology and treatment of obesity, the metabolic syndrome, and diabetes mellitus type 2. J Obes.

[44] Sattler FR, Bhasin S, He J, Yarasheski K, Binder E, Todd Schroeder E, Castaneda-Sceppa C, Kawakubo M, Roubenoff R, Dunn M, Hanh C, Stewart Y, Martinez C, Azen SP (2011). Durability of the Effects of Testosterone and Growth Hormone Supplementation in Older Community Dwelling Men: The HORMA Trial. Clin Endocrinol.

[45] Travison TG, Basaria S, Storer TW, Jette AM, Miciek R, Farwell WR, Choong K, Lakshman K, Mazer NA, Coviello AD, Knapp PE, Ulloor J, Zhang A, Brooks B, Nguyen AH, Eder R, LeBrasseur N, Elmi A, Appleman E, Hede-Brierly L, Bhasin G, Bhatia A, Lazzari A, Davis S, Ni P, Collins L, Bhasin S (2011). Clinical Meaningfulness of the Changes in Muscle Performance and Physical Function Associated With Testosterone Administration in Older Men With Mobility Limitation. J Gerontol Series A, Biol Sci Med Sci.

[46] Wang C, Ilani N, Arver S, McLachlan RI, Soulis T, Watkinson A (2011). Efficacy and safety of the 2% formulation of testosterone topical solution applied to the axillae in androgen-deficient men. Clin endocrinol.

[47] Bhasin S, Cunningham GR, Hayes FJ, Matsumoto AM, Snyder PJ, Swerdloff RS, Montori VM (2010). Testosterone therapy in men with androgen deficiency syndromes: an Endocrine Society clinical practice guideline. J Clin Endocrinol Metab.

[48] Bhasin S, Woodhouse L, Casaburi R, Singh AB, Mac RP, Lee M, Yarasheski KE, Sinha-Hikim I, Dzekov C, Dzekov J, Magliano L, Storer TW (2005). Older men are as responsive as young men to the anabolic effects of graded doses of testosterone on the skeletal muscle. J Clin Endocrinol Metab.

[49] Hyde Z, Flicker L, Almeida OP, Hankey GJ, McCaul KA, Chubb SAP, Yeap BB (2010). Low free testosterone predicts frailty in older men: the health in men study. J Clin Endocrinol Metab.

[50] Krasnoff JB, Basaria S, Pencina MJ, Jasuja GK, Vasan RS, Ulloor J, Zhang A, Coviello A, Kelly-Hayes M, D'Agostino RB, Wolf PA, Bhasin S, Murabito JM (2010). Free testosterone levels are associated with mobility limitation and physical performance in community-dwelling men: the Framingham Offspring Study. J Clin Endocrinol Metab.

[51] Srinivas-Shankar U, Roberts SA, Connolly MJ, O'Connell MDL, Adams JE, Oldham JA, Wu FCW (2010). Effects of testosterone on muscle strength, physical function, body composition, and quality of life in intermediate-frail and frail elderly men: a randomized, double-blind, placebo-controlled study. J Clin Endocrinol Metab.

[52] Sattler FR, Castaneda-Sceppa C, Binder EF, Schroeder ET, Wang Y, Bhasin S, Kawakubo M, Stewart Y, Yarasheski KE, Ulloor J, Colletti P, Roubenof R, Azen SP (2009). Testosterone and growth hormone improve body composition and muscle performance in older men. J Clin Endocrinol Metab.

[53] Auyeung TW, Lee JSW, Kwok T, Leung J, Ohlsson C, Vandenput L, Leung PC, Woo J (2011). Testosterone but not estradiol level is positively related to muscle strength and physical performance independent of muscle mass: a cross-sectional study in 1489 older men. Eur J Endocrinol/Eur Fed Endocr Soc.

[54] Chen F, Lam R, Shaywitz D, Hendrickson RC, Opiteck GJ, Wishengrad D, Liaw A, Song Q, Stewart AJ, Cummings CE, Beals C, Yarasheshki KE, Reicin A, Ruddy M, Hu X, Yates NA, Menteski J, Herman GA (2011). Evaluation of early biomarkers of muscle anabolic response to testosterone. J Cachex Sarcopenia Muscle.

[55] Frobell RB, Lohmander LS, Roos EM (2007). The challenge of recruiting patients with anterior cruciate ligament injury of the knee into a randomized clinical trial comparing surgical and non-surgical treatment. Contemp Clin Trials.

[56] Comins J, Brodersen J, Krogsgaard M (2010). Treatment for acute anterior cruciate ligament tear. N Engl J Med.

[57] Shah VM, Andrews JR, Fleisig GS, McMichael CS, Lemak LJ (2010). Return to play after anterior cruciate ligament reconstruction in National Football League athletes. Am J Sports Med.

[CR58] The pre-publication history for this paper can be accessed here:http://www.biomedcentral.com/1471-2482/14/102/prepub

